# Plasticity in timing of avian breeding in response to spring temperature differs between early and late nesting species

**DOI:** 10.1038/s41598-021-84160-6

**Published:** 2021-03-08

**Authors:** David J. Messmer, Ray T. Alisauskas, Hannu Pöysä, Pentti Runko, Robert G. Clark

**Affiliations:** 1 Department of Biology, University of Saskatchewan, 112 Science Place, Saskatoon, SK S7N 2E5 Canada; 2 Environment and Climate Change Canada, 115 Perimeter Road, Saskatoon, SK S7N 0X4 Canada; 3 Natural Resources Institute Finland (Luke), Natural Resources, Joensuu, Finland; 4 Montana Fish, Wildlife and Parks, 1420 East 6th Avenue, Helena, MT USA; 5 Hirvipurontie 44, FI-71750 Maaninka, Finland

**Keywords:** Climate-change ecology, Phenology

## Abstract

Plasticity for breeding dates may influence population vulnerability to climate change via phenological mismatch between an organism’s life cycle requirements and resource availability in occupied environments. Some life history traits may constrain plasticity, however there have been remarkably few comparisons of how closely-related species, differing in key traits, respond to common phenology gradients. We compared population- and individual-level plasticity in clutch initiation dates (CID) in response to spring temperature among five duck species with early- to late-season nesting life histories. Plasticity was strongest in females of the earliest breeding species (common goldeneye [*Bucephala clangula*], mallard [*Anas platyrhynchos*], and gadwall [*Mareca strepera*]), whereas late-nesting lesser scaup (*Aythya affinis*) and white-winged scoter (*Melanitta fusca deglandi*) did not respond. These results contrast with previous work in other bird families that suggested late-breeders are generally more flexible. Nevertheless, late-breeding species exhibited annual variation in mean CID, suggesting response to other environmental factors unrelated to spring temperature. Goldeneye and gadwall females varied in their strength of individual plasticity (‘individual × environment’ interactions) and goldeneye and scoter females showed evidence of interannual repeatability of CID. Fitness consequences of CID plasticity in response to spring phenology, including trophic mechanisms and population consequences, warrant investigation.

## Introduction

Plasticity in timing of breeding is an important factor in avian population responses to climate variability and change^1^. Advancing phenology of spring resource productivity in many parts of the northern hemisphere is one of the outcomes of recent climate change^2^, and rigidity of avian breeding dates could induce mismatches between energy and nutrient requirements and peak supply of seasonal resources^3,4^. Traits that constrain plasticity, such as migration distance, may limit the ability of birds to track changes in the phenology of their breeding environment^5,6^, with negative consequences for population resilience^7^.

Avian species in mid- to high-latitude environments have a relatively short breeding season to hatch and rear young. However, within this short window, species have distinct strategies for their relative timing of breeding. Even among closely related species, average breeding dates can be separated by weeks or months^8,9^. Whether these differences in average breeding dates constrain plasticity remains an area of active research. Among eight species of arctic-nesting shorebirds, Saalfeld and Lanctot^9^ found that while most species advanced nesting dates in response to earlier snowmelt, species that were relatively late breeders or that employed an opportunistic settling strategy seemed most capable of keeping pace with spring warming. Similarly, among three sympatric species of Antarctic penguins, Lynch et al.^10^ found that a late breeding species was more plastic to October temperatures than the earliest breeding one.

Northern hemisphere duck species have a wide range of mean nest initiation dates, with average nesting dates separated by as much as 60 days^8^. In contrast to species mentioned above, early-nesting ducks seem to adjust nesting dates more readily to match the onset of spring-like conditions, although it is uncertain whether late-nesting species respond similarly. Indeed, Saalfeld and Lanctot^9^ hypothesized that late-nesting species may be able to advance nesting dates more so than species that nest soon after their earlier arrival to breeding locations. In contrast, Gurney et al.^11^ showed that lesser scaup (*Aythya affinis*; hereafter referred to as scaup), a late-nesting species, had similar average nest initiation dates across wide latitudinal and growing season length gradients (44–65°N latitude and site-average 100–257 growing days, respectively), and showed little response to annual variation in spring phenology. Meanwhile, early-nesting species, such as mallard (*Anas platyrhynchos*) and common goldeneye (*Bucephala clangula*; hereafter referred to as goldeneye) seemed to adjust timing of nesting to match annual spring phenology cues^12–14^. Drever et al.^15^ hypothesized that populations of late-nesting duck species could be more vulnerable to advancing spring conditions if females did not adjust to warmer weather by nesting earlier. To date, the hypothesis that late-nesting species are less flexible has not been widely tested.

Here, we compare plasticity in clutch initiation dates (CID) exhibited by individually-marked females of five duck species characterized by early-, mid- and late-season nesting strategies. Specifically, we estimate population- and individual-level plasticity for breeding dates, with emphasis on response to phenology of spring warming. We also evaluate whether individuals of the same species differ in their plasticity and whether they nest consistently early or late relative to the population mean across years (i.e., repeatability). Such individual variation is a necessary precursor for genetic adaptation to climate change expected during this century^4,16,17^.

## Results

**Figure 1:**
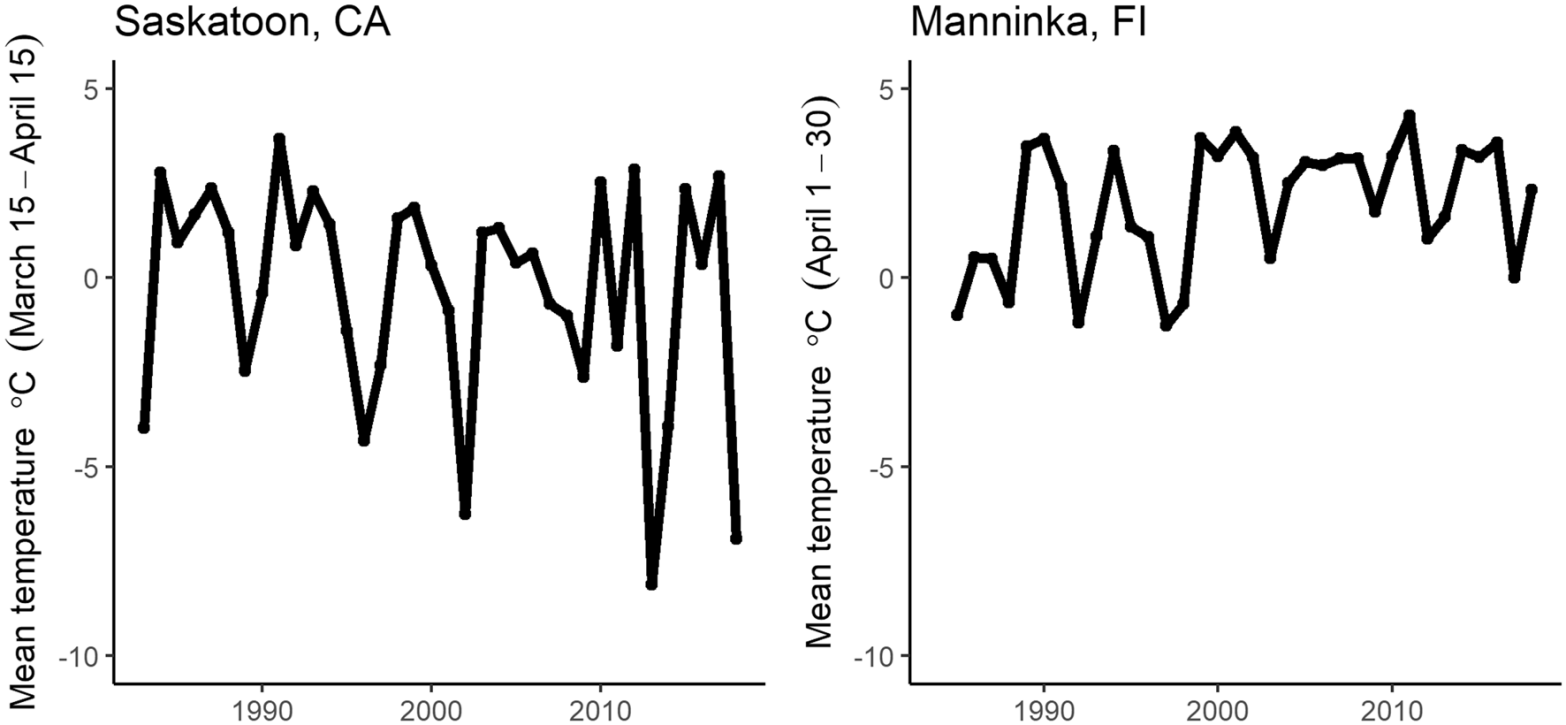
Average of mean daily spring temperatures for weather stations near the Canadian sites (Saskatoon, Saskatchewan; 1983–2018) and the Finnish site (Maaninka; 1983–2018).

**Figure 2:**
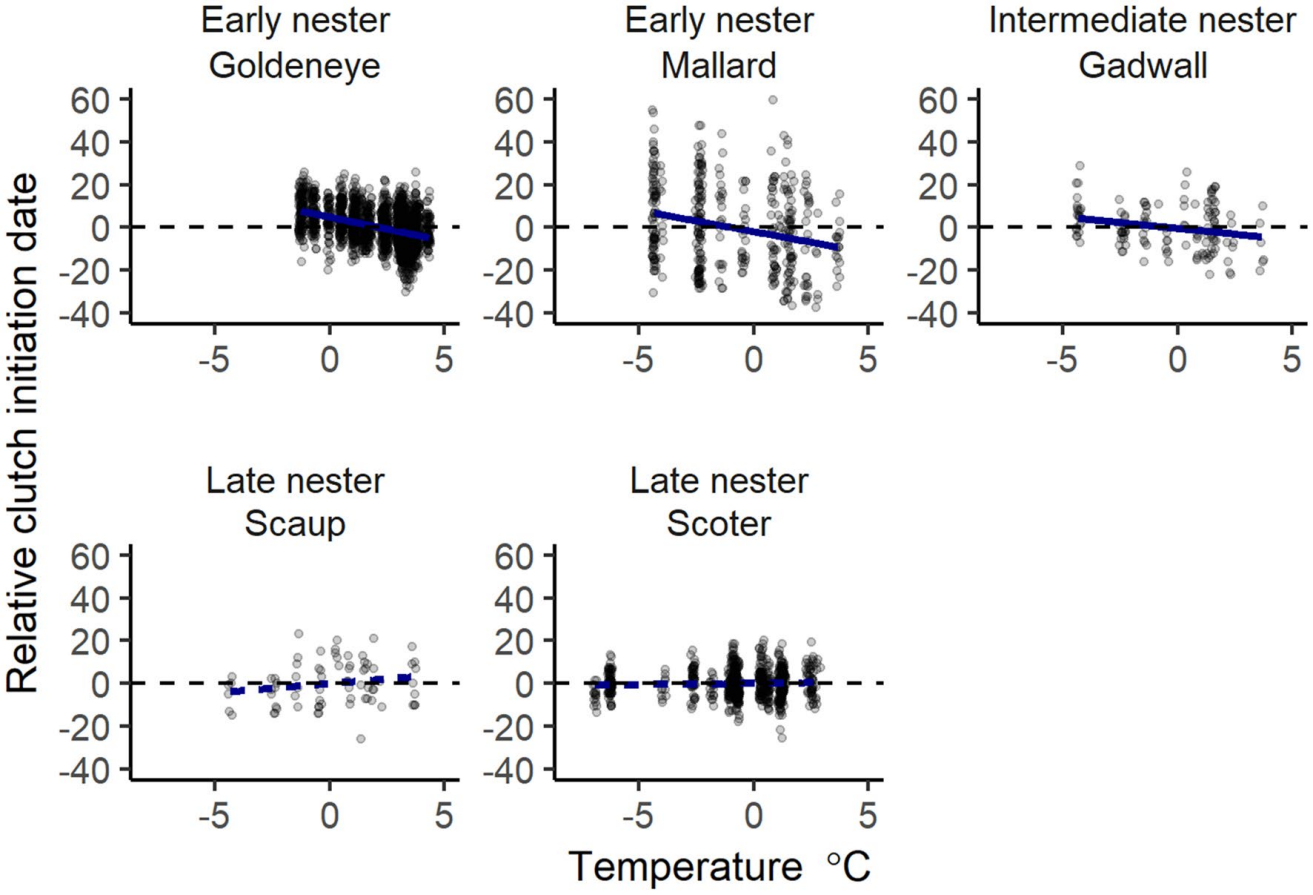
Population-level response to spring temperature by species. A regression line is included for illustrative purposes from the linear model ‘clutch initiation date ~ spring temperature’. Slope of response from full model is reported in Fig. 3, dashed lines indicate 95% confidence intervals overlapped 0.

Sample sizes of individually-marked females and nesting attempts recorded per individual between years varied by species (early-season nesting: goldeneye *n* = 567 individuals and 1989 total records, mallard *n* = 278 and 389; mid-season nesting: gadwall (*Marcera strepera*) *n* = 67 and 132; late-season nesting: scaup *n* = 27 and 73, white-winged scoter (*Melanitta fusca deglandi*; hereafter referred to as scoter) *n* = 404 and 760; Supplementary Table S1). Variation in the environmental phenology index, spring temperature, also differed between continents (Fig. 1) and within each species’ unique time series (S.D. of spring temperatures across years: goldeneye = 1.67 °C, mallard = 2.4 °C, gadwall = 2.2 °C, scaup = 2.3 °C and scoter = 3.3 °C). Scoters showed the least annual variation in CID (S.D. = 6.6 days) and mallards the greatest (S.D. = 19.9 days; Supplementary Table S1, Fig. 2). For mallards, which frequently renest after a failed breeding attempt, results below were similar regardless of the portion of late-nesting records removed to reduce impacts of renesting (up to 50%; results not shown). Likewise, results were similar for all species when data were subset to include only individuals with a minimum of 2 or 3 lifetime interannual nesting attempts (results not shown).

### Population and individual responses to spring temperature

**Figure 3:**
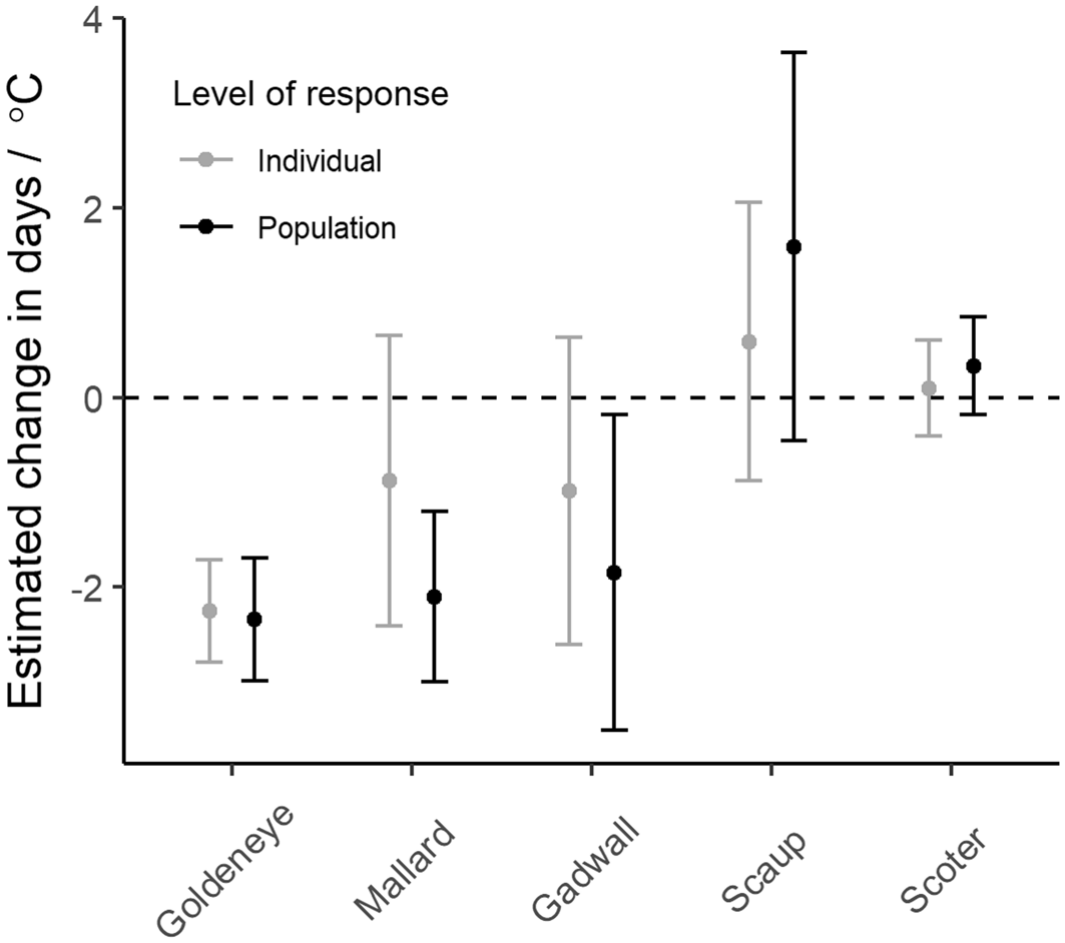
Comparison of estimates of population- and individual-level responses in clutch initiation date (95% CI) for spring temperatures. Similar estimates at both levels indicate that population-level responses are likely due to individual phenotypic plasticity. Goldeneye and mallard are early-nesters, gadwall mid-season nester, and scaup and scoter are late-nesters. Sample sizes in Supplementary Table S1.

Population response to spring temperature was most evident among early-breeding goldeneye and mallard, and in mid-season breeding gadwall (Fig. 3; complete parameter estimates shown in Supplementary Table S2, Fig. 3). We did not detect clear statistical differences in the degree of individual versus population responses, regardless of species. Mallards had the largest apparent discrepancy; however, the difference between responses was not significant at 95% confidence level (${\widehat{\beta }}_{population-individual}$ = − 1.23 ± 0.89 S.E.M.) likely because of the lack of precision in individual-level plasticity estimates. This result leaves some ambiguity about the role of individual plasticity as the mechanism for observed population-level plasticity.

**Figure 4:**
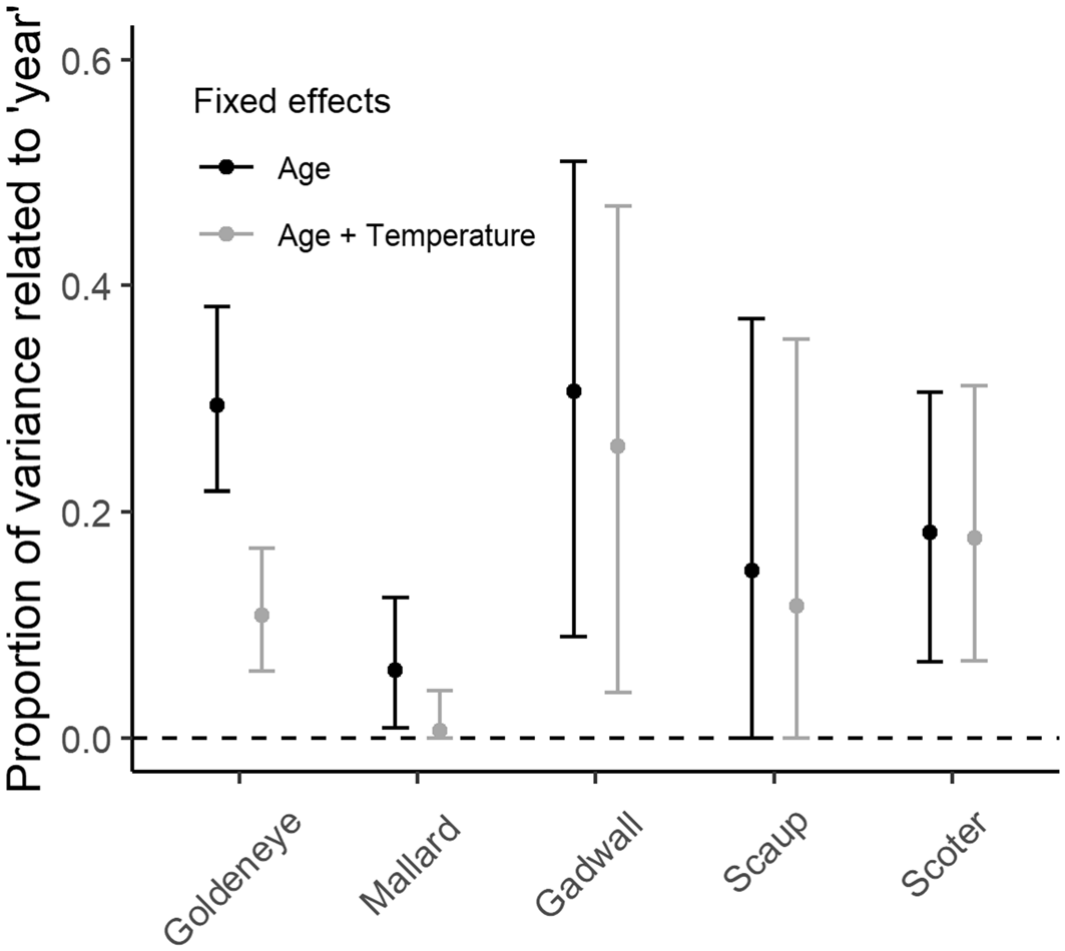
Proportion of variance in clutch initiation date related to the random effect of year for female ducks. Age was controlled for in all models, however results are shown with and without spring temperature in the fixed effects. Confidence intervals are based on parametric bootstrapping (*n* = 1000 simulations). Sample sizes and years included in each species’ time series are shown in Supplementary Table S1.

**Figure 5:**
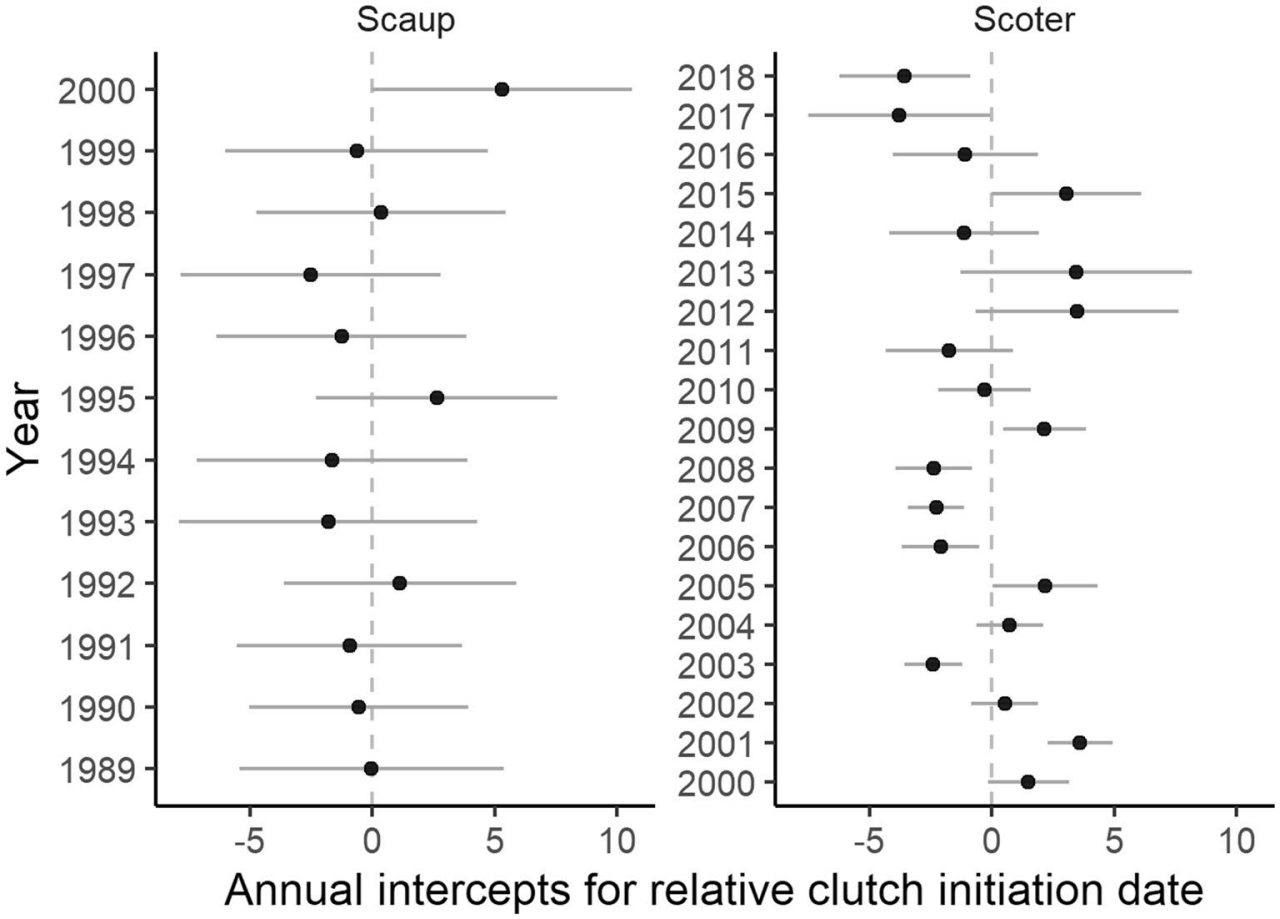
Annual deviations from the grand average relative clutch initiation date in late-breeding species which did not respond to the spring temperature. Shown are random effect estimates ± S.D. for females with ≥ 2 lifetime inter-annual nesting attempts (scaup *n* = 73 and scoter *n* = 544).

Plasticity in CID in response to mean spring temperature was not evident in late-breeding scaup or scoter for either the early season window, March 15–April 15, the late season window, May 15–June 15, or the full pre-nesting season window, March 15–June 15. However, annual variation in mean CID of scaup and scoter was confirmed by support for the ‘year’ random intercept (Table 1) and variance components analysis (Fig. 4). Thus, scaup and scoter adjusted CID to either unmeasured annual cues on the breeding grounds or other factors experienced before breeding (Fig. 5). Similarly, goldeneye and gadwall may also have responded to additional factors not fully accounted for by spring temperature (Fig. 4).

**Table 1:** Comparison of models for species-specific female plasticity in clutch initiation date. Likelihood ratio tests are used to sequentially test more complex models including random intercepts for ‘Year’ and individual females ‘I’, and random slopes for the female × spring temperature interaction ‘I × E’. Fixed effects are constant in all models, accounting for age, age^2^, and spring temperature. Estimates are based on females that made at least two inter-annual breeding attempts over their lifetime.

Species	Random effects	Log likelihood	ΔDF	Likelihood ratio	*P* value
Mallard	None	− 807.97	A		
Mallard	Year	− 805.53	1	4.90	0.027
Mallard	Year, I	− 805.51	1	0.02	0.880
Mallard	Year, I × E	− 805.47	1	0.10	0.755
Scaup	None	− 269.80	NA		
Scaup	Year	− 267.89	1	3.83	0.050
Scaup	Year, I	− 266.36	1	3.05	0.081
Scaup	Year, I × E	− 266.19	1	0.34	0.560
Gadwall	None	− 413.39	A		
Gadwall	Year	− 400.90	1	24.98	0.000
Gadwall	Year, I	− 400.24	1	1.34	0.247
Gadwall	Year, I × E	− 397.78	1	4.91	0.027
Goldeneye	None	− 6,095.60	NA		
Goldeneye	Year	− 6,031.45	1	128.30	0.000
Goldeneye	Year, I	− 5,814.46	1	433.97	0.000
Goldeneye	Year, I × E	− 5,813.21	1	2.51	0.113
Scoter	None	− 1,795.12	NA		
Scoter	Year	− 1,773.78	1	42.67	0.000
Scoter	Year, I	− 1,765.19	1	17.18	0.000
Scoter	Year, I × E	− 1,765.16	1	0.05	0.822

### Repeatability and individual variation in response to spring temperature

We found consistency in CID among female goldeneye, scoter, and possibly scaup (Table 1). For goldeneye and scoter, sample sizes were sufficient to calculate intraclass correlation coefficients (ICC), estimated as 0.32 (95% CI 0.23–0.40) and 0.15 (95% CI 0.07–0.24), respectively. We found support for individual differences in the strength of response to spring temperature only in gadwall and possibly goldeneye (‘Individual’ × ‘Environment’ [I × E] random slope supported; Table 1). Such individual heterogeneity in degree of plasticity was not detected in mallard, scaup or scoter, although relatively low sample sizes may have precluded detection in mallard and scaup^18^.

## Discussion

### Species-specific breeding date plasticity in response to spring temperature

Population-level responses to spring temperature conditions have been described for several avian species^4^ including ducks (reviewed in^12^). However, our study simultaneously compared individual- versus population-level responses across avian species with different average breeding dates. Strong evidence of plasticity in response to spring temperature was detected among the earliest breeding duck species (mallard and goldeneye) and mid-season breeding gadwall, whereas late-nesting species (scaup and scoter) did not adjust CID to variation in spring temperature phenology. Although late-breeding scoter and scaup pairs typically arrive on breeding sites much later than mallards or goldeneye, they do not nest for several weeks after arrival and presumably have time to respond to local temperature cues. However, our results were not consistent with the hypothesis that late-nesting species are more plastic to spring temperature^9^. Alternative explanations that link reduced plasticity of late nesting species to effects of migration distance or anthropogenic food subsidies seem unlikely. Other studies suggested that migration distance is unimportant to CID plasticity^19^ although CID plasticity in mallard could plausibly be facilitated by anthropogenic food subsidies acquired during spring migration^20^. However, we are unaware of any such subsidy for the highly plastic goldeneye that also nest early, like mallard. Our study design could not completely distinguish species-specific versus population- or site-specific responses, *sensu*^21^, because we lacked spatial replication within species.

While female scoter and scaup did not breed earlier in years with warmer springs there was evidence, nevertheless, for annual variation in mean CID from models with ‘year’ as a random intercept term (Table 1). This annual variation was common for all species to varying degrees, even in models that accounted for spring temperature (Fig. 4), suggesting that other factors could act as additional cues for adjustment of timing of breeding. For scoter and scaup, these cues did not appear to be related to temperatures measured later in the breeding season as might be expected given the relationship between the abundance of amphipods, an important prey and increasing water temperatures^22–24^. Compared to early nesting species, scoter and scaup might be more responsive to precipitation^25^ or possibly concealing effects of vegetation at prospective nest sites^26^.

### Do individual responses to spring temperature explain population-level plasticity?

Estimates of population- and individual-level plasticity of the early-breeding goldeneye were very similar suggesting that individual plasticity accounted for most population response to temperature phenology. However, in the other early- and mid-season breeding species, mallard and gadwall, we did not detect clear evidence of individual plasticity despite finding that their populations’ mean CID tracked spring temperature across years. Population response to spring phenology is well-documented in both mallard and gadwall^27,28^; nevertheless, we detected no individual plasticity in either species despite sample sizes of 74 mallards and 46 gadwalls with ≥ 2 between-year clutch initiation records. This inconsistency of population- and individual-level plasticity has been documented in other avian species^29,30^ and can occur when newly recruited juveniles adjust breeding or migration chronology while adults show consistent nesting dates across years^30^. However, such a phenomenon would be difficult to detect in mallard and gadwall given high variance in individual inter-year CID, and a strong tendency for juveniles to initiate clutches later than adults. Further investigation is required to understand the apparent absence of individual plasticity in these species.

### Potential fitness consequences of breeding date plasticity

Reproductive consequences of breeding date plasticity have not been fully established in most species (see^4^), including ducks. However, early-breeding goldeneye females produced more recruited offspring in early and late spring conditions than did late-breeding ones^14^, a general pattern reported in some other duck species^31^ including lesser scaup^32^. Apparent first-year survival also appears to be higher among early-hatched female mallard and gadwall ducklings (RGC, unpubl), and while first-year survival has not been quantified in white-winged scoter, ducklings from early-hatched broods survive at higher rates^33^. So, in general, the ability of breeding females to respond appropriately to early onset of spring could be advantageous. Scaup and scoter populations in particular remain below North American conservation goals, and determining how variation in spring phenology contributes to population dynamics in these species remains an important question^15,34^.

### Individual variation in breeding date plasticity and repeatability

Adaptation (i.e., microevolution) may occur in environments where prolonged changes in mean environmental phenology have been observed or are predicted. Breeding-resource mismatches that reduce individual fitness can create selection pressure to adjust timing of breeding^35^. However, for adaptation to occur, there must be a genetic basis for breeding date or phenotypic plasticity thereof. While we did not have female pedigree information to directly address this question (*sensu*^35^), our results for repeatability of CID across years and varying individual phenotypic plasticity provide starting points. Among early-breeding goldeneye and late-breeding scoter for which we had adequate sample sizes to test for repeatability, CIDs were correlated across years; this implies that that some females consistently nested at dates that differed from the population mean regardless of overall shifts in annual breeding chronology. Further, there was evidence for goldeneye that individual females varied in response to spring phenology (‘Individual × Environment’ interaction [‘I × E’]), consistent with previous findings^14^. Although sample sizes were likely too low to conduct robust tests of the ‘I × E’ interaction for mallard and scaup, we detected the interaction in gadwall. Whether differences among species and individuals in repeatability and plasticity are related to their environment, e.g., consistent wintering or migratory habitat choices versus fixed genetic traits, remains unclear. The fitness consequences of repeatability and its heritability should be topics of future research to understand how species may be able to adapt to climate change through microevolution^4^.

## Methods

### Study areas and species

Species included early-nesting goldeneye (1985–2018) and mallard (1984–1998), mid-late-nesting gadwall (1986–2002), and late-nesting scaup (1989–2000) and white-winged scoter (2000–2018). Goldeneye data were collected by PR near Maaninka in central Finland (63°09′N, 27°17′E). The study area consisted of 23 lakes and ponds and the bays of larger lakes, surrounded by agricultural land and managed forests^14,36^. White-wing scoter data were collected by RTA and associates for females nesting on islands and adjacent uplands of Redberry Lake, within the Redberry Lake Migratory Bird Sanctuary (52°41′N, 107°11′W), in southcentral Saskatchewan, Canada. The lake is at the southern edge of the scoter breeding range and is surrounded predominately by cropland and aspen parkland forest^37,38^. Data for the remaining three species were collected by RGC and associates at the 361-ha St. Denis National Wildlife Area (52°12′N, 106°5′W), Saskatchewan, Canada, located ~ 100 km southeast of the Redberry Lake site but within a similar landscape^26^.

### Clutch initiation dates

At Maaninka, goldeneyes nested in nest boxes, checked for eggs three to four times during the breeding season; hatch date was used as the index for CID in this population^14^. At Redberry Lake, islands and adjacent uplands were searched for nests on foot using trained dogs^37^, while at St. Denis, nests were found by using a combination of foot-searches and by dragging a chain between two all-terrain vehicles^26^. At both Canadian sites, nest searches were completed three to five times per year during the nesting season. CID was estimated by back-calculating the nest age from clutch size (assuming 1.5 eggs per day for scoter, and 1 egg per day for all other species) and estimated stage of embryonic development via the candling method of Weller^39^.

Many female ducks renest if their first clutch is destroyed. The influence of re-nesting on data analysis was reduced by excluding, for each species and year, dates deemed to be outliers (Tukey’s boxplot definition, > 1.5 times the interquartile range of CID above the 75th percentile). This resulted in removal of 16 goldeneye, 5 mallard, 6 gadwall, 1 scaup, and 15 scoter records. Unknown renesting females not removed by this procedure would have the effect of weakening the observed responses to spring temperature. Because mallards have a particularly high propensity to renest after nest failure^40^, we repeated tests after sequentially removing larger numbers of records of late initiated clutches—up to the 50% of the latest nesting records—to ensure results were robust.

### Spring environmental phenology

Ice-out phenology on breeding areas is an important early constraint on the duck breeding season. At northern latitudes, breeding pairs do not begin settling in breeding territories until widespread open-water conditions exist^41^. Ice-out is also the beginning of the wetland growing season at which point solar energy influx and water temperatures increase leading to increased aquatic invertebrate productivity on which females rely to rear broods^22–24^. Ice-out date has been recorded at the Maaninka site for each year of the goldeneye study and strongly influences goldeneye breeding dates^14^. However, no comparable observations of ice-out date were recorded at Canadian sites, so we used spring air temperatures as an index of ice-melt phenology based on data from the nearest meteorological stations. To create this index, we calculated long-term average (1984–2018) date at which mean daily temperature exceeded 0 °C, which was ~ April 1 at both Finnish and Canadian sites. We bounded this date by 15 days on either side (i.e., March 15–April 15), and used mean daily temperature in this window as an index to the relative annual timing of ice-out at each site. At Maaninka, mean temperatures during this time frame were correlated to ice-out dates (r = − 0.46, *p* = 0.006, 34 years) but we found the period April 1–30 had a better correspondence with the ice-out dates (r = − 0.77, *p* < 0.001, 34 years). This window was corroborated by Korhonen^42^ who found ice-out dates of Lake Kallavesi in central Finland were also correlated strongly (r = − 0.80) with mean April temperatures during 1848–2002. Therefore we used this period in the goldeneye analysis while retaining the March 15–April 15 window for Canadian sites as it was more predictive of CIDs in models below. For Maaninka, we downloaded temperature data from http://mesi.metla.fi/ (accessed February 27, 2019; see^43^). The nearest recording station for Redberry Lake and St Denis was Saskatoon, Saskatchewan (52°10′N, 106°43′W), located ~ 65 and 45 km from each study area, respectively (downloaded from http://climate.weather.gc.ca/).

For the late-breeding species, scoter and scaup, we also tested whether temperatures measured closer to the beginning of their mean nest initiation period (mean temperature May 15–June 15), or temperature measured across a wider window of the breeding season (mean temperature March 15–June 15) predicted CID better. Both species rely heavily on amphipod species (Amphipoda) for provisioning ducklings^44–46^, many of which seem to increase in biomass later in the summer in a manner related to seasonally increasing water temperatures^22–24^.

### Estimating population- and individual-level plasticity and interannual repeatability

Population- and individual-level plasticity were estimated separately for each species using a within-subject centering method in a mixed effects modeling framework^47^. In this framework, we modeled CID with 2 variables derived from spring temperature data. First, a within-subject, mean-centered temperature covariate was derived ($x_{i,t} - \overline{x}_{i}$; where subscript i refers to individual and t to year) where regression coefficients for each species represent individual-level plasticity to spring temperature. The second covariate represented between-subject variation in spring temperature ($\overline{x}_{i}$, i.e., mean temperature observation for each individual’s record) whose coefficient represents population-level plasticity^47^. Because older ducks nest earlier than younger ones^27,48–50^, we accounted for age with a quadratic term. We also included 2 random intercept terms: (1) ’year’, to account for shared but unexplained annual variation in CID, and (2) ’female id’, to account for multiple, potentially correlated, observations of CID from the same female, and to quantify the degree to which individual females consistently bred early or late relative to the overall population. The model was:

$$\begin{aligned} & CID_{i,t} = age_{i,t} + age_{i,t}^{2} + \left( {x_{i,t} - \overline{x}_{i} } \right) + \overline{x}_{i} + year_{t} + female_{i} + \varepsilon_{i,t} \\ & year_{t} \sim N\left( {0, \sigma_{year}^{2} } \right) \\ & female_{i} \sim N\left( {0, \sigma_{female}^{2} } \right) \\ & \varepsilon_{i,t} \sim N\left( {0, \sigma_{residual}^{2} } \right) \\ \end{aligned}$$

For species where within- and between-subject parameters seemed to differ, we tested statistical support for the difference by fitting a model with the non-within-subject centered spring temperature variable ($x_{i,t}$) and the between-individual mean $(\overline{x}_{i} )$ where we can then interpret the slope for $\overline{x}_{i}$ as the difference between population- and individual-level plasticity^47^. Because species may respond to annual cues other than spring temperature or may be impacted by shared exogenous factors outside the breeding area, plasticity was assessed more generally by using a likelihood ratio test to evaluate support for a ‘year’ random intercept. We calculated the proportion of variance in CID associated with ‘year’ as $\sigma_{year}^{2} /\left( {\sigma_{year}^{2} + \sigma_{female}^{2} + \sigma_{residual}^{2} } \right)$ from variances estimated in the model above (i.e., accounting for age and female-specific intercepts). High variance attributable to ‘year’ suggests that females respond to annual factors other than spring temperature. For reference, we also calculated the proportion of variance explained by year without the fixed effect of spring temperature, i.e., the total proportion of variance attributable for year. We calculated 95% CI for these variance components using parametric bootstrapping over 1000 simulated datasets^51^.

We calculated the adjusted intraclass correlation coefficient (ICC), a repeatability statistic^52,53^, for the two species with adequate sample sizes, goldeneye and scoter. The ICC describes the percentage of phenotypic variation observed in the population attributable to individuals that consistently nested early or late relative to the population mean, conditional on spring temperature. We included data from all females with nests detected in ≥ 2 years; results using a more restrictive inclusion criterion of ≥ 3 between-year breeding attempts produced very similar results (results not shown).

We tested for significant differences in female response to spring temperature (frequently referred to as ‘Individual × Environment’ interaction [I × E]) by comparing a random slope model to the random intercept-only model, following Charmantier et al.^54^. We made this comparison for all species, although statistical power to detect the interaction was likely low for scaup, gadwall, and mallard because of limited sample sizes (Supplementary Table S1), whereas goldeneye and scoter tests were likely more robust^18^. We censored females with only a single recorded between-year nesting attempt in these models to avoid overparameterization (i.e., more random effects than observations). All models were fit in program R 3.5.2^55^ using the package lme4 1.1–20^56^ and restricted maximum likelihood estimation.

### Ethics

Handling and marking of all animals conformed with the laws and regulations of Canada and Finland. Research at Redberry Lake (Protocol Number 20000010) and St. Denis were approved by the University of Saskatchewan Animal Research Ethics Board and Wildlife Research Permits issued by Environment and Climate Change Canada.

## Supplementary Information

Supplementary Tables.

Supplementary Data 1.

Supplementary Data 2.

Supplementary Code 1.

Supplementary Code 2.

Supplementary File.

## Data Availability

All data analyzed during this study and code for analysis and figures are included in this published article (and its Supplementary Information files).
