# Supplementary material for: Plasticity in timing of avian breeding in response to spring temperature differs between early and late nesting species
Source: Sci Rep. 2021 Mar 8;11:5410. doi: 10.1038/s41598-021-84160-6 (PMC7940653; doi:10.1038/s41598-021-84160-6)
Supplement: Supplementary file 1 — Supplementary Tables. [file 41598_2021_84160_MOESM1_ESM.pdf]

Plasticity in timing of avian breeding in response to spring temperature differs between early and late nesting species

Running heading: Plasticity in timing of avian breeding

David J. Messmer[1*], Ray T. Alisauskas[1,2], Hannu Pöysä[3], Pentti Runko[3] and Robert G. Clark[1,2].

[1] Department of Biology, 112 Science Place, University of Saskatchewan, Saskatoon, Saskatchewan S7N 2E5, Canada.

[2] Environment & Climate Change Canada, 115 Perimeter Road, Saskatoon, Saskatchewan S7N 0X4, Canada.

[3] Natural Resources Institute Finland (Luke), Natural Resources, Joensuu, Finland

*Correspondence to: [dmessmer@gmail.com]; present address: *Montana Fish, Wildlife and Parks, 1420 East 6th Avenue, Helena, MT*

Electronic Supplemental Material:

Supplementary Table S1. Descriptions of study site locations, study years, and clutch initiation date for five duck species (s.d. =standard deviation). Also shown are sample sizes of individually-marked female ducks in our study individuals with >1 recorded lifetime interannual nest attempts. While hatch dates are used as the indicator of goldeneye breeding date in analysis, clutch initiation dates are shown here for comparison by back-calculating initiation date as 'hatch date – (1.5 × 8 + 28)', where eight is the mean clutch size and 28 the mean incubation length for goldeneye.

Species[a]	Study Site	Years	Average clutch initiation date	Total females in study	Females with >1 nesting attempt
Common goldeneye	Maaninka, Finland	1985-2018	4 May S.D. = 8.7	567	390
Mallard	St. Denis, Canada	1983-1998	20 May S.D. = 19.9	278	74
Gadwall	St. Denis, Canada	1986-2001	6 June S.D. = 10.6	67	46
Lesser scaup	St. Denis, Canada	1989-2000	15 June S.D. = 10.2	27	27
White-winged scoter	Redberry Lake, Canada	2000-2018	17 June, S.D=6.6	404	188

[a] Common goldeneye (*Bucephala clangula*), mallard (*Anas platyrhynchos*), gadwall (*Mareca strepera*), lesser scaup (*Aythya affinis*), white-winged scoter (*Melanitta fusca deglandi*).

Supplementary Table S2. Parameter estimates from the maximal linear mixed model of CID (Julian date) with 1) additive fixed effects terms for age, age^2, within-subject centered spring temperature, within-subject mean spring temperature, and 2) random intercepts for female ID, year, and a random slope for within-subject centered spring temperature by female ID.

Species	Effect Type	Group	Term	Estimate	S.E.M.	Lower 95% CI	Upper 95% CI
Gadwall	Fixed	NA	Intercept	155.56	1.87	151.9	159.22
Gadwall	Fixed	NA	age	-11.45	8.12	-27.36	4.47
Gadwall	Fixed	NA	age^2	-1.3	8.27	-17.5	14.91
Gadwall	Fixed	NA	Within-subject centered temperature	-1.09	0.9	-2.87	0.68
Gadwall	Fixed	NA	Within-subject mean temperature	-1.76	1.01	-3.75	0.22
Gadwall	Random	Female ID	S.D. of random intercept	4.18	NA	NA	NA
Gadwall	Random	Female ID	S.D. of random slope for within-subject centered temperature	1.48	NA	NA	NA
Gadwall	Random	year	S.D. of random intercept	5.83	NA	NA	NA

Gadwall	Random	Residual	S.D. of residual error	6.95	NA	NA	NA
Goldeneye	Fixed	NA	Intercept	159.56	0.49	158.6	160.52
Goldeneye	Fixed	NA	age	-121.93	6.93	-135.52	-108.34
Goldeneye	Fixed	NA	age^2	89.93	5.73	78.7	101.16
Goldeneye	Fixed	NA	Within-subject centered temperature	-2.23	0.27	-2.77	-1.69
Goldeneye	Fixed	NA	Within-subject mean temperature	-2.16	0.4	-2.95	-1.37
Goldeneye	Random	Female ID	S.D. of random intercept	4.41	NA	NA	NA
Goldeneye	Random	Female ID	S.D. of random slope for within-subject centered temperature	0.25	NA	NA	NA
Goldeneye	Random	year	S.D. of random intercept	2.36	NA	NA	NA
Goldeneye	Random	Residual	S.D. of residual error	5.12	NA	NA	NA
Mallard	Fixed	NA	Intercept	136.38	1.41	133.61	139.15
Mallard	Fixed	NA	age	2.57	19.98	-36.59	41.73
Mallard	Fixed	NA	age^2	66.33	19.85	27.42	105.23
Mallard	Fixed	NA	Within-subject	-0.7	0.81	-2.3	0.89

			centered temperature				
Mallard	Fixed	NA	Within-subject mean temperature	-2.76	0.88	-4.48	-1.05
Mallard	Random	Female ID	S.D. of random intercept	0.86	NA	NA	NA
Mallard	Random	Female ID	S.D. of random slope for within-subject centered temperature	0.04	NA	NA	NA
Mallard	Random	year	S.D. of random intercept	0	NA	NA	NA
Mallard	Random	Residual	S.D. of residual error	19.16	NA	NA	NA
Scaup	Fixed	NA	Intercept	165.72	1.77	162.25	169.18
Scaup	Fixed	NA	age	11.06	10.85	-10.2	32.32
Scaup	Fixed	NA	age^2	4.63	10.67	-16.29	25.55
Scaup	Fixed	NA	Within-subject centered temperature	0.52	0.8	-1.04	2.09
Scaup	Fixed	NA	Within-subject mean temperature	1.74	1.07	-0.36	3.83
Scaup	Random	Female ID	S.D. of random intercept	4.83	NA	NA	NA

Scaup	Random	Female ID	S.D. of random slope for within-subject centered temperature	0.76	NA	NA	NA
Scaup	Random	year	S.D. of random intercept	3.75	NA	NA	NA
Scaup	Random	Residual	S.D. of residual error	8.22	NA	NA	NA
Scoter	Fixed	NA	Intercept	167.32	0.84	165.68	168.97
Scoter	Fixed	NA	age	-0.69	7.66	-15.7	14.32
Scoter	Fixed	NA	age^2	3.99	6.32	-8.4	16.39
Scoter	Fixed	NA	Within-subject centered temperature	-0.03	0.29	-0.6	0.53
Scoter	Fixed	NA	Within-subject mean temperature	-0.01	0.37	-0.74	0.72
Scoter	Random	Female ID	S.D. of random intercept	2.63	NA	NA	NA
Scoter	Random	Female ID	S.D. of random slope for within-subject centered temperature	0.2	NA	NA	NA
Scoter	Random	year	S.D. of random intercept	3.06	NA	NA	NA

Scoter	Random	Residual	S.D. of residual error	5.5	NA	NA	NA
